# Editorial: Development of nanotherapeutics with multi-functionalities for targeting cancer cells

**DOI:** 10.3389/fimmu.2024.1380937

**Published:** 2024-02-22

**Authors:** Houria Boulaiz

**Affiliations:** ^1^ Department of Anatomy and Human Embryology, Faculty of Medicine, University of Granada, Granada, Spain; ^2^ Biosanitary Institute of Granada (ibs.GRANADA), Granada, Spain; ^3^ Biopathology and Regenerative Medicine Institute (IBIMER), Granada, Spain

**Keywords:** cancer, smart nanoparticles, immunotherapy, nanomedicine, nanotherapy

Despite advances in early detection and classical therapies, cancer remains the second leading cause of death worldwide (1). This is probably because cancer is a dynamic disease and tumor cells generally become more heterogeneous during their development, acquiring more resistance to treatments over time. In addition, the tumor microenvironment (TME) consists of a heterogeneous population, involving a variety of infiltrating and resident host cells, stem cells, endothelial cells, cancer-associated fibroblasts and immune system cells, that plays a critical role in the pathogenesis of cancer (2). Adding further complexity to this pathology, the tumor set and its microenvironment both vary during the development of the disease. Thus, sequencing analysis demonstrated that the genetic architecture of the same tumor is temporally heterogeneous (3). Moreover, tumor heterogeneity has a profound impact on the immune microenvironment and it has been statistically demonstrated that the magnitude of intratumoral genetic heterogeneity correlates with the heterogeneity of immune cell infiltration, implying the co-evolution of tumor genetic architecture and immune microenvironment (4, 5). Hence, the development of innovative targeted cancer therapies, which are more effective and less aggressive, that improve the patient’s quality of life is of great interest. Nanotechnology for targeted cancer therapy represents a promising approach that uses nano-sized therapeutic materials as potent anticancer agents, yielding encouraging outcomes in both research and clinical trials. The current Research Topic highlights a diverse panel of reviews and two original research papers on the development of nanotherapeutics with multi-functionalities for targeting cancer cells.

In the field of immunotherapy, there are various strategies based on the use of smart nanoparticles (NPs) aiming to boost the immune system to detect and eliminate tumors as well as the tumor microenvironment (TME) cells. In this context, Kousar et al. used CD44 for targeting the delivery of oncolytic Newcastle disease virus encased in thiolated chitosan for prolonged release in cervical cancer. With this approach, authors achieved an active targeting while concealing the virus from the immune system and also ensuring a sustained release of the virus in the TME over an extended period. Another strategy was provided by Yang et al. that has used self-assembled albumin NPs to trigger pyroptosis, fostering synergistic photodynamic, photothermal, and immune therapies in triple-negative breast cancer. In addition, the nanovaccine, a vaccine employing nanotechnology to transport antigens and adjuvants to immune cells, is gaining prominence as a promising approach for cancer immunotherapy. Its capacity to trigger immune responses and induce tumor-specific immunity underscores its potential effectiveness. Yao et al. discussed the compositions and types of nanovaccine, and the mechanisms behind their anti-tumor effects based on the latest research.

Tumor development is a long and complex process, and monotherapies are susceptible to immune tolerance or unwanted effects, often benefiting only a limited population. Consequently, combination therapy is considered an interesting approach to increase the efficacy of antitumor treatments. The use of nano-vectors for drug delivery could be a perfect ally for conventional therapy by enhancing treatment efficacy and improving patient outcomes. In this context, prostate-specific membrane antigen (PSMA), a type II transmembrane protein found in 88% of patients with recurrent Prostate cancer (PCa), is a marker used to direct the NPs to the Prostate tumor. PSMA, also expressed in the neovascular system during malignant angiogenesis, has proven to be a promising tumor associated antigen (TAA) and is engaged in multimodal combination therapy of PCa. He et al. explore the potential advantages and challenges of the diverse applications of PSMA-targeted NPs in the advanced PCa management from a cross-disciplinary perspective. Moreover, autophagy, a vital cellular degradation process, closely linked to cancer development has undergone various strategic approaches. He et al. reviews recent achievements focusing on the use of diverse nanocarriers for delivering autophagy regulators and inhibitors, enhancing chemotherapy drug efficacy by reducing breakdown in cancer cells. Targeting TME, Yang et al. provide a concise overview of the distinctive characteristics of the TME and the latest developments in metal NPs designed to respond to TME for immunotherapy and explore the potential, challenges and prospects of combining NPs with other treatments, including chemotherapy, radiotherapy, and photodynamic therapy.

Altogether, this Research Topic addresses the challenges and prospects of nanotechnology to improve the efficacy of anti-tumor immunotherapy, while analyzing the potential side effects and risks associated with the use of nanotherapeutics. There is no doubt that the efforts made in recent years in innovating the design of NPs have led to an encouraging result in experimental studies of tumor immunotherapy, which will certainly play a crucial role in the future of antitumor treatment strategies. However, due to its high economic cost, much work remains to be done before this technology reaches the patient. Cost and clinical translation considerations must be taken into account. The success of the clinical translation of smart nanomaterials requires close interdisciplinary collaboration involving not only basic researchers but also specialists in different fields such as immunology, oncology, and the pharmaceutical industry among others, so that cancer patients can benefit.

## Author contributions

HB: Conceptualization, Writing – original draft, Writing – review & editing.
